# The Role of *TLR2*, *TLR4* and *CD14* Genetic Polymorphisms in Gastric Carcinogenesis: A Case-Control Study and Meta-Analysis

**DOI:** 10.1371/journal.pone.0060327

**Published:** 2013-04-02

**Authors:** Natalia Castaño-Rodríguez, Nadeem O. Kaakoush, Khean-Lee Goh, Kwong Ming Fock, Hazel M. Mitchell

**Affiliations:** 1 School of Biotechnology and Biomolecular Sciences, The University of New South Wales, Sydney, Australia; 2 Department of Medicine, Faculty of Medicine, University of Malaya, Kuala Lumpur, Malaysia; 3 Division of Gastroenterology, Department of Medicine, Changi General Hospital, Singapore, Singapore; University of California Merced, United States of America

## Abstract

**Background:**

In addition to *Helicobacter pylori* infection, host genetic factors contribute to gastric cancer (GC). Recognition of *H. pylori* is known to involve Toll-like receptors (TLR), which subsequently leads to activation of NF-*κ*B. Thus, the overall aim of this study was to estimate for the first time the pooled effect size of polymorphisms in *TLR2*, *TLR4* and *CD14* on GC development through a meta-analysis.

**Methods:**

A case-control study comprising 284 ethnic Chinese individuals (70 non-cardia GC cases and 214 functional dyspepsia controls) was conducted for the genotyping of *TLR2* -196 to -174del, *CD14* -260 C/T and *TLR4* rs11536889 using PCR, RT-PCR and mass spectrometry. Case-control studies of *TLR2*, *TLR4* and *CD14* polymorphisms and GC were searched up to June 2012. Pooled odds ratios and 95% confidence intervals were obtained by means of the random effects model.

**Results:**

In our ethnic Chinese case-control study, the *TLR4* rs11536889 C allele increased the risk of GC (OR: 1.89, 95%CI: 1.23–2.92) while the *CD14* -260 T allele was protective (OR: 0.62, 95%CI: 0.42–0.91). *TLR2* -196 to -174 increased the risk of GC only in *H. pylori*-infected individuals (OR: 3.10, 95%CI: 1.27–7.60). In the meta-analysis, *TLR4* Asp299Gly showed borderline results in the general analysis (pooled OR: 1.58, 95%CI: 0.98–2.60), nevertheless, stratified analysis by ethnicity showed that the mutant allele was a definitive risk factor for GC in Western populations (pooled OR: 1.87, 95%CI: 1.31–2.65). There was a potential association between the *TLR2* -196 to -174 deletion allele and GC in Japanese (pooled OR: 1.18, 95%CI: 0.96–1.45). *TLR4* Thr399Ile did not provide significant results.

**Conclusions:**

*TLR4* rs11536889 and *CD14* -260 C/T are associated with non-cardia GC in Chinese. Based on our meta-analysis, the TLR signalling pathway is involved in gastric carcinogenesis, *TLR4* Asp299Gly and *TLR2* -196 to -174del showing associations with GC in an ethnic-specific manner.

## Introduction

Despite a major decline in incidence and mortality rates over several decades, gastric cancer (GC) still remains a major cause of morbidity and mortality worldwide [1]. According to global cancer statistics, 934,000 new cases were diagnosed in 2002, which represents 8.6% of all cancers worldwide [2]. Almost two thirds of GC cases occur in East Asia, Eastern Europe and Central and South America, these regions being classified as high risk-GC populations (age-standardised rates in men >20 per 100,000) [3]. The incidence of GC in Chinese individuals resident in China represents 42% of the above worldwide estimation, with Chinese ethnicity identified as an independent risk factor for the development of GC in multiracial studies [2], [3], [4].

Chronic inflammation has been associated with an increased risk of developing several human malignancies, including GC [5]. In 1988, Correa proposed a human model of intestinal-type gastric carcinogenesis [6]. The model hypothesised a sequence of events progressing from inflammation to atrophy, to metaplasia, to dysplasia, to carcinoma *in situ*, and finally to invasive GC. Although *Helicobacter pylori* has been established as the most important aetiological risk factor for GC, the pathogenesis of GC involves the combined effects of bacterial, host and environmental factors [7], [8], [9], [10], [11]. Given that *H. pylori* is initially targeted by the toll-like receptors (TLR) signalling pathway, it is conceivable that functionally relevant polymorphisms in genes of this arm of the immune system could affect the magnitude and direction of the host response against the infection.

The involvement of the TLR signalling pathway in infectious, autoimmune and inflammatory diseases is well accepted [12]. TLR are pattern recognition receptors (PRR) of the innate immune system that recognise a wide variety of molecules. TLR4 was initially identified as the potential signalling receptor for *H. pylori* on gastric epithelial cells [13]. After forming a complex with the LPS-binding protein (LBP), *H. pylori* lipopolysaccharide (LPS) interacts with the monocyte differentiation antigen CD14 (CD14) [14]. Together with TLR4, this complex induces the TLR4-mediated MyD88-dependent signal transduction pathway, which leads to the activation of transcription factors, mainly NF-*κ*B, and cytokines such as TNF-α, IL-1β, IL-6 and IL-12 [12]. Although most studies conclude that TLR4 is the first innate immune response against the microorganism, others argue that TLR2 is the initial barrier against infection [15], [16], [17]. Furthermore, an interesting publication by Smith et al. showed that *H. pylori* LPS functions as a classic TLR2 ligand and induces a discrete pattern of chemokine expression in epithelial cells which involves the modulation of the expression of signalling protein tribbles 3 (TRIB3), a molecule that has been implicated in the regulation of NF-*κ*B [18]. Fortunately, a very recent study by Yokota et al. seems to provide a possible explanation for this dilemma [19]. Yokota et al., not only showed that *H. pylori* LPS is initially targeted by TLR2 as described by others, but, for the first time, showed that this TLR2 activation leads to cell proliferation and TLR4 expression via the ERK1/2-ERK1/2 kinases (MEK1/2) pathway [19]. The final outcome of this signalling pathway is increased proliferation of gastric epithelial cells and the instauration of a strong inflammatory reaction. Interestingly, these authors also proposed that *H. pylori* can enhance inflammatory reactions mediated by TLR4 agonists such as other bacterial LPS which could contribute to gastric inflammation and carcinogenesis [19].

*TLR2* is located in the long arm of chromosome 4 comprising two 5′ non-coding exons followed by a third coding exon. A polymorphism that causes a 22-bp deletion, known as *TLR2* -196 to -174del, has been shown to influence the promoter activity of *TLR2*. This polymorphism has not only been associated with GC but also other inflammation-related cancers including gallbladder, bladder, prostate and cervical cancers [20], [21], [22], [23], [24].

*TLR4* is located in chromosome 9q32–q33 and contains 4 exons. Polymorphisms in *TLR4* have been extensively studied in several populations in an attempt to find associations with diverse pathologies such as cancer, atherosclerosis and infectious diseases [25], [26], [27]. Among these, *TLR4* Asp299Gly (rs4986790) and Thr399Ile (rs4986791) are located in the coding sequence resulting in amino acid substitutions that affect the TLR4 extracellular domain. Another functionally relevant polymorphism, identify as *TLR4* rs11536889, showed a novel association with periodontitis in Japanese individuals [28]. This mutation is located in the 3′untranslated region (UTR) and it is believed to influence transcription and/or translation [28].

*CD14* is encoded by a single-copy gene located at bands 5q23–q31 that consists of approximately 3900 bp harboured in two exons. The most studied polymorphism in this gene involves a C-T substitution at base-pair 260 of the 5′flanking region mainly known as *CD14* -260 C/T (rs2569190) but also called *CD14* -159 C/T [29]. Le Van and collaborators investigated the molecular basis for the effects of this polymorphism on CD14 regulation in monocytes and hepatocytes and showed that the *CD14* -260 T allele had decreased affinity for DNA/protein interactions at a GC box containing a binding site for stimulatory protein (SP) 1, SP2 and SP3 transcription factors [30]. In addition, reporter gene assays demonstrated that monocytic cells showing low levels of SP3, which inhibits activation by SP1 and SP2, have increased transcriptional activity of the T allele [30]. Therefore, the authors concluded that the SP3:SP1+SP2 ratio might play an important mechanistic role in regulating *CD14* transcription and in determining the differential activity of the two variants of the *CD14* promoter [30].

Given the importance of TLRs in regulation of the innate immune response to *H. pylori* infection, in the current study, we conducted a case-control study and meta-analysis assessing *TLR2* -196 to -174del, *TLR4* Asp299Gly, *TLR4* Thr399Ile, *TLR4* rs11536889 and *CD14* -260 C/T and their association with gastric carcinogenesis, in an attempt to clarify the limited and current conflicting evidence, and to establish the true impact of the TLR signalling pathway in this pathology. To date, two meta-analyses have been published on *TLR4* polymorphisms and risk of cancer in general [27], [31], but no meta-analyses have been published addressing the association between these and the other selected polymorphisms in the current study and the risk of GC in particular.

## Materials and Methods

### Case-control Study in Ethnic Chinese

#### Ethics Statement

This study was approved by the Human Ethics Committee (HREC) of the University of New South Wales (HREC 08115 and HERC 02144). Written informed consent was obtained from each individual recruited to the study.

### Patients and Methods

All subjects were ethnic Chinese individuals presenting for upper gastrointestinal endoscopy at the Department of Medicine, University Hospital of Malaysia (Kuala Lumpur) and The Changi General Hospital (Singapore). Patients known to be infected with the Human Immunodeficiency Virus or who had been prescribed non-steroidal anti-inflammatory drugs (NSAIDs), anti-microbial agents or acid suppressants (H_2_ receptor antagonists and proton pump inhibitors) in the two-month period prior to recruitment were excluded.

Seventy patients newly diagnosed with a primary non-cardia GC (International Classification of Diseases, 9^th^ revision, code 151) based on histological confirmation and recruited during the period January 2004 to April 2007, were invited to participate in the study. The control group comprised 214 individuals diagnosed with functional dyspepsia (FD) over the same period. FD was defined as pain or discomfort centred in the upper abdomen without any identifiable organic disease, in accordance with Rome II classification system [32]. Additional information of this study sample has been described previously [33].

Genomic DNA was extracted from peripheral whole blood samples using the QIAamp^®^ Blood DNA Mini Kit as described by the manufacturer (Qiagen; Hilden, Germany). DNA was rehydrated in sterile water and normalised to 10 ng/ µl for customised SNP genotyping of *TLR4* rs11536889 through the application of matrix assisted laser desorption ionisation time-of-flight (MALDI-TOF) mass spectrometry, the Sequenom MassARRAY iPLEX™ assay (San Diego, CA, USA [34], [35], at the Australian Genome Research Facility Ltd, St Lucia, University of Queensland, Australia. Genotyping of *TLR2* -196 to -174del polymorphism was conducted by standard PCR using a pair of primers designed by Tahara et al. [24]. Genotyping of *CD14* -260 C/T was performed using real time-PCR. Hairpin primers were designed based on the methodology published by Hazbon et al. and Chan et al. [36], [37]. The primer sequences and thermal cycling conditions for genotyping of *TLR2* -196 to -174del and *CD14* -260 C/T are outlined in Table 1.

**Table 1:** PCR primer sequences and thermal conditions used for genotyping of *CD14* and *TLR2* polymorphisms in gastric cancer patients and functional dyspepsia controls.

Polymorphism	Molecular Technique	Primer	Sequence	Product (bp)	Tm^a^	Tm^b^	Tm^c^	Cycles
*CD14*-260 C/T*	Real-time PCR	Forward 1Forward 2Reverse	5′-TCC CAT GTT TCA GAG AGG GGG A-3′5′-CCC CAT GTT TCA GAG AGG GGG G-3′5′-TGC CAG GAG ACA CAG AAC-3′	73	Stage 2: 95°C for 15 sStage 3: 95°C for 15 s	Stage 2: 69°C for 20 sStage 3: 60°C for 30 s	Stage 2: 72°C for 20 sStage 3: 72°C for 10 s	1040
*TLR2* -196 to -174 del	PCR	ForwardReverse	5′-CAC GGA GGC AGC GAG AAA-3′ 5′-CTG GGC CGT GCA AAG AAG-3′	Insertion: 286Deletion:264	95° C for 30 s	62°C for 30 s	72°C for 1 min	35

Tm^a^: Denaturation temperature, Tm^b^: Annealing temperature, Tm^c^: Extension temperature. *The primers were designed using the reverse (bottom) strand.

We have previously genotyped *TLR4* Asp299Gly (*TLR4* Asp299Gly and Thr399Ile are in linkage disequilibrium) in a subset of the same ethnic Chinese population. The methodology and results have been published elsewhere [38].

The presence of *H. pylori* IgG antibodies in GC patients and FD controls was determined by means of an in-house enzyme-linked immunosorbent assay (ELISA), previously described by Mitchell et al. [39], which had high sensitivity and specificity in a Chinese population. In addition to ELISA, serum samples from GC patients shown to be *H. pylori* negative by ELISA were investigated further by Immunoblot (MPD Helico Blot 2.1, MP Biomedicals, Australia) according to the manufacturer's instructions. These results have been published in part elsewhere [38].

### Meta-analysis

#### Literature Search Strategy

Electronic databases (PUBMED, Scopus, Science Direct, Ovid, Biosis Previews, Scirus databases, Ingenta, Proquest, Informaworld, Nature Publishing Group, CINAHL, IMBIOMED, Scielo and LILACS) were searched up to June 2012 for all case-control studies evaluating *TLR2*, *TLR4* and *CD14* polymorphisms and risk of GC in humans. The search strategy included Medical Subject Headings (MeSH) terms and text words as described in Table 2. In addition, hand-searching was also conducted. No other limits or special features (e.g. explosion) were employed.

**Table 2:** Medical subject headings and text words used in the literature search strategy.

Gene	MeSH terms and text words
*CD14*	“Antigens, CD14” [Majr] OR “CD14” AND “Polymorphism, Genetic” [Mesh] AND “Stomach Neoplasm” [Mesh] OR “gastric cancer”
*TLR4*	“Toll-Like Receptor 4/genetic” [Mesh]) OR “TLR4” AND “Stomach Neoplasm” [Mesh] OR “gastric cancer”
*TLR2*	“Toll-Like Receptor 2” [Mesh]) OR “TLR2” AND “Stomach Neoplasm” [Mesh] OR “gastric cancer”

MeSH: medical subject headings, NA: not available.

#### Study Selection

Studies were selected if they met the following conditions: diagnosis of primary GC based on histological assessment, the study had a case-control design, the selected polymorphisms in the control subjects satisfied the Hardy Weinberg equilibrium (HWE), it supplied sufficient information to calculate the OR and the manuscript was published as a full paper in a peer-reviewed journal, not as an abstract or similar type of summary.

#### Data Extraction

The following information was collected from each study: author, year of publication, journal, study population, tumour location (cardia and non-cardia GC), molecular technique, *H. pylori* status (where available), total cases, total controls and number of individuals harbouring each specific allele.

### Statistical Analysis

In our case-control study, deviation from HWE was tested using the chi-square goodness-of-fit test (χ^2^). The direct count method was used to estimate the genotype and allele frequencies. The odds ratios (OR) and 95% confidence intervals (CI) were calculated by means of the Fisher's exact probability test (two-tailed *p*-values). The data was analysed by means of the programs GraphPad Prism version 5.02 (GraphPad Software Inc; San Diego, USA) and SPSS version 19.0.0 (SPSS Inc; Chicago, USA).

For the meta-analysis, the allele frequencies from individual studies were calculated as the number of cases or controls harbouring at least one allele divided by the total number of chromosomes included in each of the corresponding groups. The pooled ORs were calculated by weighting individual ORs by the inverse of their variance. For each polymorphism, this pooled OR as well as the 95% CI were obtained by means of both the fixed and the random effects model. The random effects model was chosen as it assumes that there is a distribution of true effect sizes rather than one true effect, and it assigns a more balanced weight to each study. Heterogeneity was calculated by means of the Cochrańs *Q* test (*P*-value <0.10 is indicative of heterogeneity). Due to the low power of this statistical test when meta-analysis includes a small number of studies, the Higginś test (*I* ^2^) was also used to measure the degree of inconsistency in the results of the studies and to describe the percentage of total variation across studies due to heterogeneity rather than chance. The terms low, moderate and high were assigned to *I* ^2^ values of 25%, 50% and 75%, respectively [40]. Sensitivity (one study removed) and stratified analyses by ethnic group, were conducted to assess the sensitivity of the results and to detect the possible causes of heterogeneity between studies. To assess publication bias, Funnel plots and the Egger's regression asymmetry tests were used. Data was analysed using the program Comprehensive Meta-Analysis version 2 (Biostat, Englewood, NJ, 2004).

## Results

### Ethnic Chinese Case-control Study

Demographics including age, gender and *H. pylori* status of the 284 subjects included in our ethnic Chinese case-control study are outlined in Table 3.

**Table 3:** Clinical characteristics of gastric cancer patients and functional dyspepsia controls in our ethnic Chinese case-control study.

Characteristic	GC cases (N = 70)	FD controls (N = 214)
Median age ± SD (years)	65.8±13.8	54.2±13.0
Male/Female N (%)	42/28 (60/40)	95/119 (44/56)
*H. pylori* positive N (%)	56 (80)	134 (63)

GC: gastric cancer, FD: functional dyspepsia, N: total number, SD: standard deviation.

The median age of GC patients was significantly higher than that of FD controls (65.8 vs 54.2, *P*-value: <0.0001), despite matching for age (±10 years, mean difference 10 years, *P*-value: <0.001).

Male gender was found to be more predominant in GC patients than in FD controls, showing a positive association with the development of GC in this ethnic Chinese population (OR: 1.61, 95% CI: 1.06–2.44).

*H. pylori* infection was found to be present in (67%) of individuals in this study (56/70 GC patients and 134/214 FD controls) (Table 3). Comparison of the prevalence of *H. pylori* infection in GC patients and FD controls showed that *H. pylori* infection was a risk factor for the development of GC (OR: 2.14, 95% CI: 1.23–3.70).

All polymorphisms genotyped in our case-control study were found to be in HWE in the control group showing non-significant χ^2^ values (less than 3.84). Allele and genotype frequencies as well as ORs and 95% CIs are shown in Table 4. The *TLR4* rs11536889 C allele and CC genotype markedly increased the risk of GC in these ethnic Chinese individuals showing ORs of 1.89 (95% CI: 1.23–2.92) and 5.29 (95% CI: 1.71–16.34), respectively. Individuals harbouring the *CD14* -260 T allele (OR: 0.62, 95% CI: 0.42–0.91) and TT genotype (OR: 0.37, 95% CI: 0.16–0.82) were found to be significantly protected against the development of GC. Although the *CD14* -260 CT genotype showed a tendency towards protection against GC, this was not statistically significant (OR: 0.66, 95% CI: 0.34–1.31). No significant association was found between *TLR2* -196 to 174del and GC in our case-control study.

**Table 4:** Association between *TLR2*, *TLR4* and *CD14* polymorphisms and risk of gastric cancer in ethnic Chinese individuals.

Polymorphism	Genotype/Allele	Cases (N = 70)	Controls (N = 214)	OR (95% CI)	*P*-value*
*TLR2* -196 to 174del ^†^	ins/insins/deldel/delinsdel	263678850	1019318295129	11.50 (0.84–2.68)1.51 (0.57–4.00)11.30 (0.87–1.95)	0.1900.4300.208
*TLR4* rs11536889 ^‡^	GGGCCCGC	322889244	12770632482	11.59 (0.88–2.85)5.29 (1.71–16.34)11.89 (1.23–2.92)	0.1310.0040.005
*CD14* -260 C/T	CCCTTTCT	1838147466	3410872176252	10.66 (0.34–1.31)0.37 (0.16–0.82)10.62 (0.42–0.91)	0.2820.0210.018

OR: odds ratio, CI: confidence intervals, ins: insertion, del: deletion. *Fisher's exact test two-tailed *P*-value. ^†^ This polymorphism was genotyped only in 69 GC patients and 212 FD controls. ^‡^ This polymorphism was genotyped only in 68 GC patients and 203 FD controls.

Given that *H. pylori* is known to be a major risk factor associated with GC, further analyses were performed to assess the joint effect of *TLR2* -196 to -174del, *TLR4* rs11536889 and *CD14* -260 C/T and *H. pylori* infection on risk of GC. First, to confirm that no association existed between *TLR2* -196 to -174del, *TLR4* rs11536889 and *CD14* -260 C/T and risk of *H. pylori* infection, univariate statistical analyses were conducted, showing non significant results (Table 5). Second, statistical analyses were performed to assess not only the presence or absence of the selected polymorphisms but also *H. pylori* status in relation to the risk of GC, in an attempt to determine the existence of biological interaction in the form of synergism or antagonism (Table 6). Individuals harbouring the *TLR4* rs11536889 T allele and infected with *H. pylori* were found to be at most risk of developing GC (OR: 9.75, 95% CI: 2.77–34.34). In addition, the *TLR2* -196 to -174 deletion allele increased the risk of GC only in individuals infected with *H. pylori* (OR: 3.10, 95% CI: 1.27–7.60). In contrast, *CD14* -260 C/T analyses did not provide significant results (OR: 1.78, 95% CI: 0.37–8.56).

**Table 5:** Association between *TLR2*, *TLR4* and *CD14* polymorphisms and risk of *Helicobacter pylori* infection in ethnic Chinese individuals.

Polymorphism	Genotype/Allele	*H. pylori* positive (N)	*H. pylori* negative (N)	OR (95%CI)	*P*-value^*^
*TLR2* -196 to 174del ^†^	ins/insins/deldel/delinsdel	819019252128	4639613151	11.31 (0.78–2.21)1.80 (0.67–4.82)11.31 (0.89–1.92)	0.35340.35620.2084
*TLR4* rs11536889 ^‡^	GGGCCCGC	110621028282	5036313642	10.78 (0.46–1.33)1.52 (0.40–5.75)10.94 (0.62–1.44)	0.41470.75660.8278
*CD14* -260 C/T	CCCTTTCT	419755179207	11493171111	10.53 (0.25–1.12)0.48 (0.21–1.06)10.74 (0.52–1.06)	0.11450.08570.1038

OR: odds ratio, CI: confidence intervals, ins: insertion, del: deletion. ^*^ Fisher's exact test two-tailed *P*-value. ^†^ Genotyping information available for only 281 individuals. ^‡^ Genotyping information available for only 271 individuals.

**Table 6:** Effect of *Helicobacter pylori* infection and *TLR2*, *TLR4* and *CD14* polymorphisms on gastric cancer risk.

HP status	*CD14* -260 C/T	Cases	Controls	OR	95% CI	*P*-value*
HP (−)	CC	2	9	1		
HP (−)	T carrier	9	71	0.57	0.11–3.10	0.6173
HP (+)	CC	16	25	2.88	0.55–15.09	0.2914
HP (+)	T carrier	43	109	1.78	0.37–8.56	0.7289
HP status	*TLR4* rs11536889	Cases	Controls	OR	95% CI	P-value
HP (−)	GG	3	47	1		
HP (−)	C carrier	8	31	4.04	0.99–16.44	0.053
HP (+)	GG	28	81	5.42	1.56–18.79	0.0043
HP (+)	C carrier	28	45	9.75	2.77–34.34	<0.0001
HP status	*TLR2* -196 to -174del	Cases	Controls	OR	95% CI	P-value
HP (−)	ins/ins	7	39	1		
HP (−)	deletion carrier	4	41	0.54	0.15–2.00	0.5223
HP (+)	ins/ins	19	62	1.71	0.66–4.44	0.3612
HP (+)	deletion carrier	39	70	3.10	1.27–7.60	0.0121

HP: *Helicobacter pylori*, OR: odds ratio, CI: confidence intervals. ^*^ Fisher's exact test two-tailed *P*-value.

Data on *TLR4* Asp299Gly and Thr399Ile in our ethnic Chinese case-control study has been published previously [38], therefore the results of that study were included in our meta-analysis.

### Studies Included in the Meta-analysis

**Figure 1:**
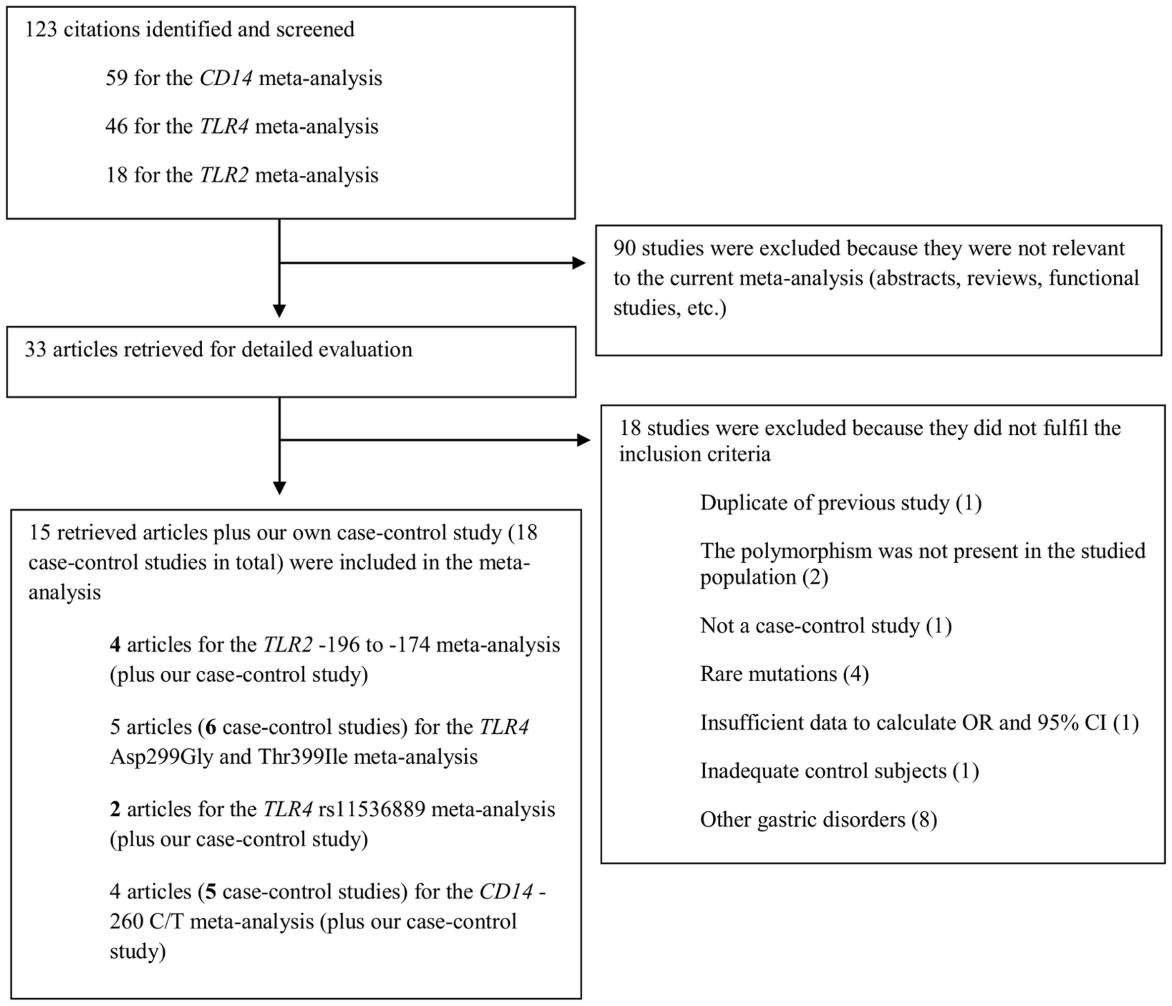
Flow diagram of included/excluded studies.

Based on our literature search strategy, 33 studies investigating the association between *TLR2*, *TLR4* and *CD14* polymorphisms and risk of GC were identified for potential inclusion in the meta-analysis (Figure 1). Of these, 18 studies, including our case-control study, fulfilled the inclusion criteria after detailed evaluation. Five of these studies were included in the *TLR2* -196 to -174del meta-analysis [24], [41], [42], [43], six in the *TLR4* Asp299Gly meta-analysis [38], [41], [44], [45], [46], three in the *TLR4* Thr399Ile meta-analysis [41], [44], [46], three in the *TLR4* rs11536889 meta-analysis [47], [48] and six in the *CD14* -260 C/T meta-analysis [49], [50], [51], [52]. Two of the articles by Hold et al. [45], [49] included in the current meta-analyses were each divided into two separate studies according to the geographical location of the subjects. Therefore, Hold et al.-1 and -2 refer to Polish subjects and to subjects from the United States, respectively. The general characteristics of our ethnic Chinese case-control study and the other studies included in the meta-analysis are outlined in Table 7. The present meta-analysis met the PRISMA (Preferred Reporting Items for Systematic Reviews and Meta-analysis) statement requirements (Checklist S1).

**Table 7:** Individual studies included in the meta-analysis of *TLR2*, *TLR4* and *CD14* polymorphisms and gastric cancer.

Author	Year	Reference	Population	Location^*^	Cases	Controls	Total	Polymorphism		OR (95% CI)^†^
Castaño-Rodriguez et al.	2012	Current study	Chinese	Non-cardia	69^‡^	212^‡^	281	*TLR2* -196 to -174 del		1.30 (0.87–1.95)
de Oliveira and Silva	2012	41	Brazilian	NA	174	225	399	*TLR2* -196 to -174 del		1.95 (1.38–2.74)
Zeng et al.	2011	43	Chinese	NA	248	496	744	*TLR2* -196 to -174 del		0.71 (0.56–0.89)
Hishida et al.	2010	42	Japanese	NA	583	1097^£^	1680	*TLR2* -196 to -174 del		1.08 (0.93–1.26)
Tahara et al.	2007	24	Japanese	Non-cardia	289	455^§^	744	*TLR2* -196 to -174 del		1.34 (1.07–1.67)
Total					1364	2487	3851	*TLR2* -196 to -174 del		
de Oliveira and Silva	2012	41	Brazilian	NA	174	225	399	*TLR4* Asp299Gly	2.68 (1.24–5.81)	
								*TLR4* Thr399Ile	1.97 (0.69–5.57)	
Schmidt et al.	2011	38	Chinese	Non-cardia	60	162	222	*TLR4* Asp299Gly	0.15 (0.02–1.15)	
Santini et al.	2008	46	Italian	NA	171	151	322	*TLR4* Asp299Gly	1.05 (0.46–2.37)	
								*TLR4* Thr399Ile	3.90 (1.30–11.72)	
Hold et al.-1	2007	45	Caucasian^¶^	Non-cardia	312	419	731	*TLR4* Asp299Gly	2.45 (1.58–3.82)	
Hold et al.-2	2007	45	Caucasian^Δ^	Non-cardia	184	211	395	*TLR4* Asp299Gly	1.85 (1.01–3.40)	
Garza-Gonzalez et al.	2007	44	Mexican	Non-cardia	78	236^¥^	314	*TLR4* Asp299Gly	1.07 (0.41–2.77)	
								*TLR4* Thr399Ile	0.23 (0.03–1.76)	
Castaño-Rodriguez et al.	2012	Current study	Chinese	Non-cardia	68^¤^	203^¤^	271	*TLR4* rs11536889	1.89 (1.23–2.92)	
Kupcinskas et al.	2011	48	Caucasians	NA	113	236	349	*TLR4* rs11536889	1.03 (0.62–1.71)	
Hishida et al.	2009	47	Japanese	NA	583	1056^≠^	1639	*TLR4* rs11536889	0.99 (0.84–1.16)	
Total					979	1404	2383	*TLR4* Asp299Gly		
					423	612	1035	*TLR4* Thr399Ile		
					766	1506	2272	*TLR4* rs11536889		
Castaño-Rodriguez et al.	2012	Current study	Chinese	Non-cardia	70	214	284	*CD14* -260 C/T	0.62 (0.42–0.91)	
Hold et al.-1	2009	49	Caucasian^¥^	Non-cardia	327	389	716	*CD14* -260 C/T	1.09 (0.89–1.35)	
Hold et al.-2	2009	49	Caucasian^ Δ^	Non-cardia	184	211	395	*CD14* -260 C/T	0.85 (0.64–1.12)	
Tahara et al.	2007	50	Japanese	Non-cardia	143	94	237	*CD14* -260 C/T	0.71 (0.49–1.02)	
Zhao et al.	2007	52	Chinese	NA	470	470	940	*CD14* -260 C/T	1.26 (1.04–1.52)	
Wu et al.	2006	51	Chinese	Non-cardia	204	210	414	*CD14* -260 C/T	0.98 (0.75–1.29)	
Total					1398	1588	2986	*CD14* -260 C/T		

NA: not available, OR: odds ratio, CI: confidence intervals. ^*^ GC location according to human stomach anatomy. ^†^ OR and 95% CI correspond to *TLR2*- 196 to -174del, *TLR4* Asp299Gly, *TLR4* Thr399Ile, *TLR4* rs11536889 or *CD14* -260 C/T allele analysis. ^‡^ This polymorphism was genotyped only in 69 gastric cancer patients and 212 functional dyspepsia controls. ^£^ 539 subjects were excluded from the control group since they presented atrophic gastritis. ^§^ Non-cancer patients (individuals with normal gastric appearance, gastritis, gastric ulcers and peptic ulcers) and healthy individuals were included in the control group. ^¶^ The study population is from Poland. ^Δ^ The study population is from the United States. ^¥^ Twenty three individuals were excluded from the initial control group (N = 259) since they had atrophic gastritis and intestinal metaplasia. ^¤^ This polymorphism was genotyped only in 68 gastric cancer patients and 203 functional dyspepsia controls. ^≠^ 536 subjects were excluded from the control group since they presented atrophic gastritis.

### Publication Bias

Funnel plots (not shown) and Eggeŕs regression tests showed that no publication bias existed in the following meta-analyses: 1) *TLR2* -196 to -174del and risk of GC (t-value: 0.91, degrees of freedom (df): 3, *P*-value: 0.429), 2) *TLR4* Thr399Ile and risk of GC (t-value: 2.00, df: 1, *P*-value: 0.294), and 3) *TLR4* rs11536889 and risk of GC (t-value: 0.90, df: 1, *P*-value: 0.532). In contrast, potential publication bias was observed in the meta-analyses of *CD14* -260 C/T and *TLR4* Asp299Gly and risk of GC (the t-value was 5.72 (df: 4, *P*-value: 0.004) and 3.52 (df: 4, *P*-value: 0.02) for *CD14* -260 C/T and *TLR4* Asp299Gly, respectively).

### Association between *TLR2* -196 to -174del and Risk of Gastric Cancer

**Figure 2:**
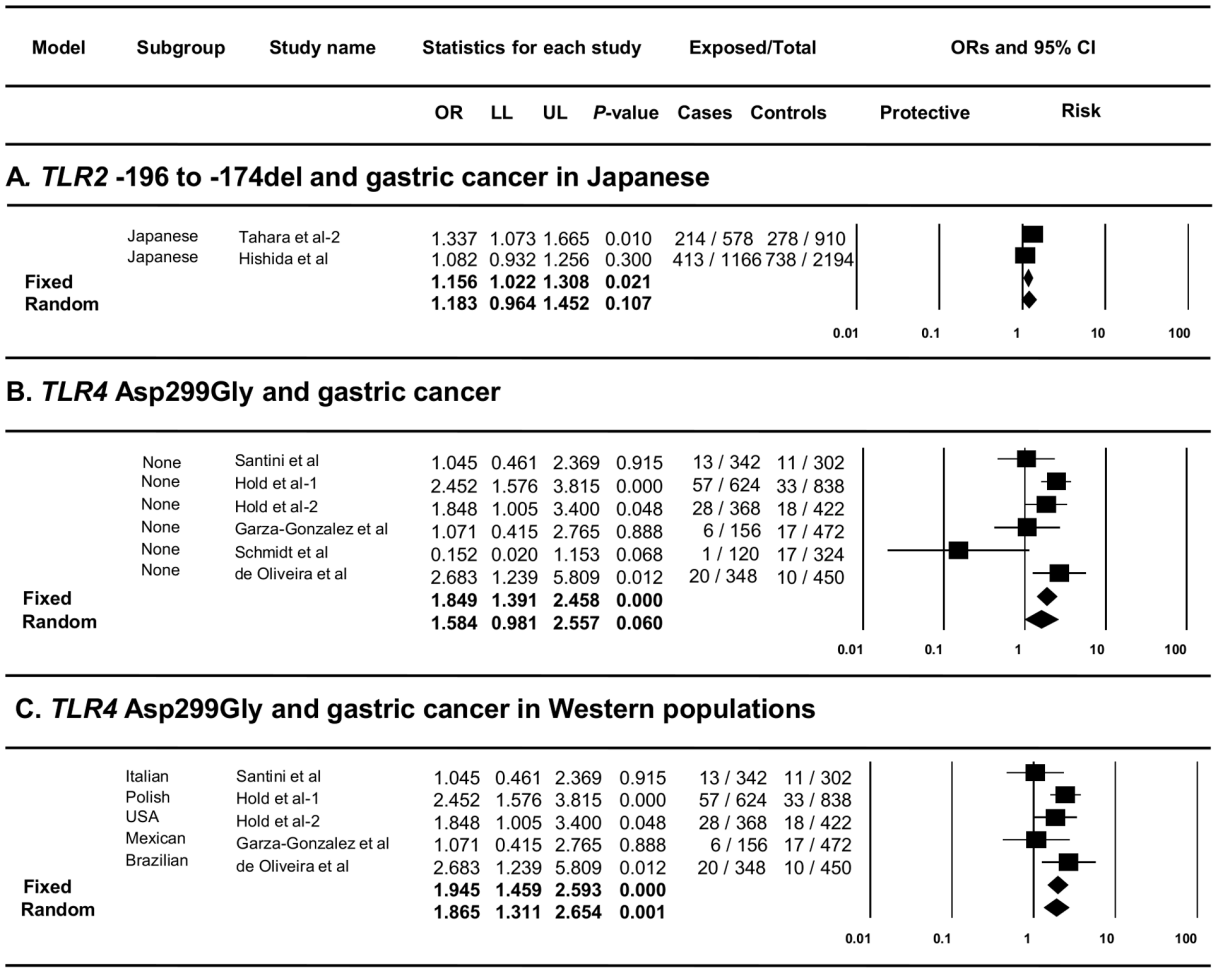
Forest plots of the meta-analysis of *TLR2* and *TLR4* polymorphisms and risk of gastric cancer. Forest plots for the meta-analysis of **A)** *TLR2* -196 to -174 del and risk of GC in Japanese, **B)** *TLR4* Asp299Gly and risk of GC, and **C)** *TLR4* Asp299Gly and risk of GC in Western populations. The pooled odds ratios with 95% confidence intervals were calculated using both the fixed and the random effects models (diamonds). The filled squares represent the studies in relation to their weights. OR: odds ratio, LL: lower limit of the 95% confidence intervals, UL: upper limit of the 95% confidence intervals.

For the meta-analysis of *TLR2* -196 to -174del and risk of GC, 1364 GC patients and 2487 controls were included. The general analysis showed a potential association to exist between *TLR2* -196 to -174del and risk of GC (pooled OR: 1.23, 95% CI: 0.89–1.70). After performing a stratified analysis by ethnicity an increased risk was observed in Japanese individuals (pooled OR: 1.18, 95% CI: 0.96–1.45) although did not reach significance (Figure 2A). Interestingly, when a sensitivity analysis (one study removed) was conducted, in which the study by Zeng et al. [43] was excluded, a significant association was observed between *TLR2* -196 to -174del and risk of GC (pooled OR: 1.41, 95% CI: 1.05–1.90). Heterogeneity was high in the general analysis (*Q*: 32.28, *P*-value: <0.001, *I^2^*: 87.61%) and moderate in the stratified analysis (*Q*: 2.43, *P*-value: 0.119, *I^2^*: 58.77%).

### Association between *TLR4* Polymorphisms and Risk of Gastric Cancer

Meta-analysis of the 6 case-control studies (979 GC patients and 1404 controls) included in the *TLR4* Asp299Gly analysis showed a borderline association with GC (pooled OR: 1.58, 95% CI: 0.98–2.56) (Figure 2B). In addition, stratified analysis by ethnicity showed the *TLR4* Asp299Gly G allele to be a significant risk factor for GC in Western populations (pooled OR: 1.87, 95% CI: 1.31–2.65) (Figure 2C). Low to moderate heterogeneity was detected in the *TLR4* Asp299Gly meta-analyses (*Q*: 11.44, *P*-value: 0.043, *I^2^*: 56.29% and *Q*: 5.48, *P*-value: 0.241, *I^2^*: 27.04% for the general and stratified by ethnicity analyses, respectively).

The meta-analysis of *TLR4* Thr399Ile included three studies comprising 423 GC patients and 612 controls. Both the general (pooled OR: 1.56, 95% CI: 0.43–5.69) and stratified by ethnicity (pooled OR: 0.81, 95% CI: 0.10–6.45 in Mestizos) analyses failed to show a statistically significant association between this polymorphism and the risk of GC. In contrast, sensitivity analysis showed significant results after excluding the study by Garza-Gonzalez et al. [44] (pooled OR: 2.72, 95% CI: 1.27–5.79). Heterogeneity was moderate in the general analysis (*Q*: 5.76, *P*-value: 0.056, *I^2^*: 65.28%) and high in the stratified analysis (*Q*: 3.39, *P*-value: 0.066, *I^2^*: 70.52%).

Meta-analysis of *TLR4* rs11536889, including 766 GC patients and 1506 controls, showed no significant associations between this polymorphism and GC in the general analysis (pooled OR: 1.21, 95% CI: 0.81–1.82) or when stratified by ethnicity (pooled OR: 1.32, 95% CI: 0.70–2.49 in Asians). Heterogeneity was high in these analyses (*Q*: 7.66, *P*-value: 0.022, *I^2^*: 73.89% and *Q*: 7.64, *P*-value: 0.006, *I^2^*: 86.92% in the general and stratified by ethnicity analyses, respectively).

### Association between *CD14* -260 C/T and Risk of Gastric Cancer

General analysis, which included data from all the studies (1398 GC patients and 1588 controls), failed to show any association between *CD14* -260 C/T and the risk of GC (pooled OR: 0.93 95% CI: 0.76–1.13). Similarly, a further stratified analysis based on ethnicity did not provide significant results (pooled OR: 0.89 95% CI: 0.64–1.22 and pooled OR: 0.98 95% CI: 0.77–1.25 in Asians and Caucasians, respectively). However, borderline results were obtained after performing sensitivity analysis, in which the study by Zhao et al. [52] was excluded (pooled OR: 0.87 95% CI: 0.72–1.05). High heterogeneity was observed in the *CD14* -260 C/T meta-analysis (*Q*: 16.77, *P*-value: 0.005, *I^2^*: 70.18% for the general analysis).

### Association between *TLR2*, *TLR4* and *CD14* Polymorphisms and Risk of *Helicobacter pylori* Infection

**Figure 3:**
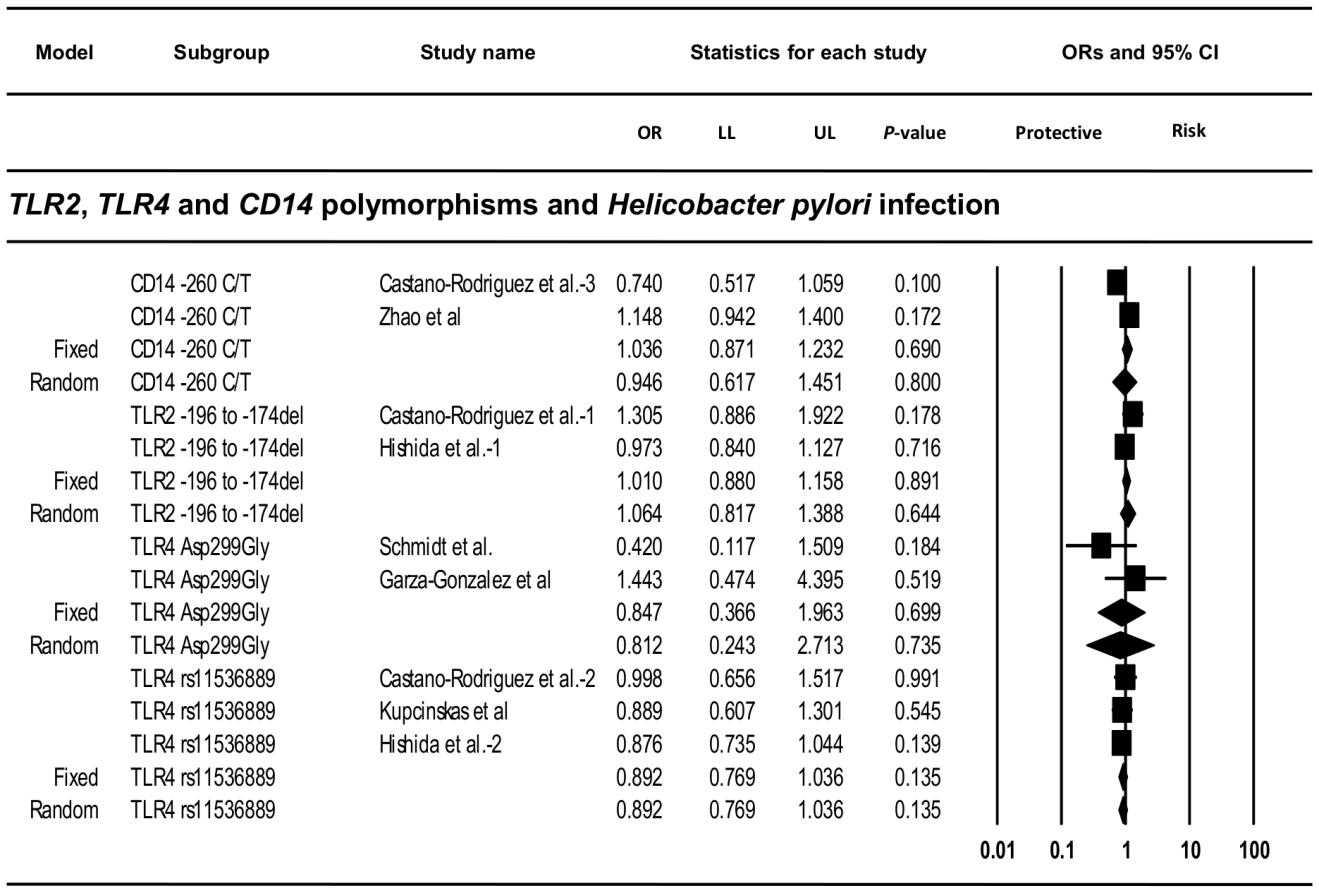
Forest plots of the meta-analysis of *TLR2*, *TLR4* and *CD14* polymorphisms and risk of *Helicobacter pylori* infection. Forest plots for the meta-analysis of *TLR2* -196 to -174del, *TLR4* Asp299Gly, *TLR4* rs11536889 and *CD14* -260 C/T and risk of *H. pylori* infection. The pooled odds ratios with 95% confidence intervals were calculated using both the fixed and the random effects models (diamonds). The filled squares represent the studies in relation to their weights. OR: odds ratio, LL: lower limit of the 95% confidence intervals, UL: upper limit of the 95% confidence intervals.

In order to determine the association between *TLR2* -196 to -174del, *TLR4* Asp299Gly, *TLR4* Thr399Ile, *TLR4* rs11536889 and *CD14* -260 C/T and *H. pylori* infection, we performed a meta-analysis with only those case-control studies that provided *H. pylori* status in relation to the genotype. Six studies, including our ethnic Chinese case-control study, provided sufficient data to calculate the OR and 95% CIs [38], [42], [44], [48], [52]. No statistically significant associations were found (Figure 3). Diverse heterogeneity was observed (*Q*: 1.93, *P*-value: 0.165, *I^2^*: 48.15%; *Q*: 2.03, *P*-value: 0.154, *I^2^*: 50.83%; *Q*: 0.32, *P*-value: 0.854, *I^2^*: 0% and *Q*: 4.42, *P*-value: 0.036, *I^2^*: 77.35% for *TLR2* -196 to -174del, *TLR4* Asp299Gly, *TLR4* rs11536889 and *CD14* -260 C/T, respectively).

## Discussion

According to extensive epidemiological evidence, *H. pylori* is a risk factor for the development of non-cardia GC [8], [10], [11]. Current evidence would suggest that *H. pylori* is initially recognised by TLR2 and 4, members of the TLR signalling pathway, which initiate an innate immune response in the host. Thus, we postulated that polymorphisms in *TLR2*, *TLR4* and *CD14*, genes that encode key components of the TLR signalling pathway, might affect the magnitude of the host response against *H. pylori* infection leading to severe long-term outcomes such as GC. Given that available studies assessing the association between genetic polymorphisms of molecules involved in the TLR signalling pathway and risk of GC were limited and conflicting, we conducted a global meta-analysis to increase the statistical power of our case-control study, to detect the pooled effect size and its magnitude, and to identify potential causes for the previous conflicting findings in other studies. General, sensitivity and stratified analyses were conducted in the current meta-analysis of *TLR2* -196 to -174del, *TLR4* Asp299Gly, *TLR4* Thr399Ile, *TLR4* rs11536889 and *CD14* 260 C/T polymorphisms and the risk of GC.

Our ethnic Chinese case-control study failed to showed an association with *TLR2* -196 to -174del. Consistent with these findings, meta-analysis of *TLR2* -196 to -174del and risk of GC showed that only in Japanese individuals *TLR2* -196 to -174del might be a risk factor for GC. Further case-controls studies are required to establish if this association is present in other ethnic groups including Caucasians. Given that TLR2 is known to stimulate the release of IL-8 in response to *H. pylori* infection [53], [54], [55], it is possible that in subjects carrying the *TLR2* -196 to -174 del/del genotype, which has been associated with decreased transcriptional activity [56], [57], IL-8 production might be reduced. Further functional studies are required to assess this question.

*TLR4* polymorphisms provided interesting findings. Although the general analysis of *TLR4* Asp299Gly, which included data from all studies, only showed borderline results, stratified analysis by ethnicity indicated that the *TLR4* Asp299Gly G allele noticeably increased the risk of developing GC in the subgroup of individuals who belonged to Western populations. The fact that *TLR4* Asp299Gly has been shown to disrupt the normal structure of the extracellular domain of TLR4, which has the potential to reduce responsiveness to *H. pylori* by diminishing the binding affinity of the bacterial ligands, increases the importance of our findings [58]. It has been suggested that this alteration might be responsible for prolonged infection and the subsequent chronic inflammation of the gastric mucosa, which has been related to an imbalance of pro- and anti-inflammatory cytokines [58]. Interestingly, Higgins et al. have shown that in TLR4-defective mice severe tissue destruction occurs due to an inadequate production of IL-10 [59]. This finding is consistent with the fact that IL-10 inhibits the production of IL-12 by activated macrophages and dendritic cells, a cytokine known to induce local production of IFN-γ from NK cells and T lymphocytes and to stimulate the subsequent adaptive immune system directing it towards a Th1 response and subsequent inflammation [60].

While *TLR4* Thr399Ile failed to show a significant association with GC in the general analysis, after performing sensitivity analysis, in which the study by Garza-Gonzalez et al. [44] was excluded, significant results were found. Sensitivity analysis is an important tool in meta-analysis when studies of doubtful eligibility, as well as poor quality or outlier studies (studies showing very different results compared to the rest of the studies), may be present. Nevertheless, given the results obtained in our meta-analysis, which only included studies conducted in Western populations, and the low frequency of this polymorphism in Asian populations [61], [62], it may be concluded that *TLR4* Thr399Ile is not involved in gastric carcinogenesis.

Although our meta-analysis did not provide significant results for *TLR4* rs11536889, our ethnic Chinese case-control study showed that individuals harbouring the *TLR4* rs11536889 C allele and CC genotype were at an increased risk of developing GC. The finding that this specific polymorphism is only a risk factor for GC in Chinese individuals is consistent with other studies where a number of polymorphisms investigated in Asian and Caucasian individuals have shown different risk associations with GC according to ethnicity [63], which potentially could explain the variability in GC incidence between races. *TLR4* rs11536889 is located in the centre of the 2818-bp *TLR4* 3′UTR and therefore, may affect mRNA stability [64]. Interestingly, this polymorphism has been shown not only to be relevant to gastric pathologies but also to other inflammation-related cancers. For example, two studies have shown a statistically significant association to exist between *TLR4* rs11536889 and the risk of prostate cancer [64], [65].

*CD14* -260 C/T showed a statistically significant association with GC in our ethnic Chinese case-control study. The results showed that the *CD14* -260 T allele and TT genotype conferred protection against the disease in these subjects. In the meta-analysis, while the general and stratified by ethnicity analyses showed that the association between *CD14* 260 C/T and the development of GC was not statistically significant, sensitivity analysis excluding the study by Zhao et al. [52], the only study that did not specify tumour location, provided borderline results inferring that the *CD14* -260 T allele might be a protective factor against the development of non-cardia GC mainly, the main GC subtype in East Asian populations including our ethnic Chinese population. *CD14* -260 C/T involves a C-T substitution at base-pair 260 of the 5′flanking region where the promoter, other enhancers and protein binding sites are likely to be located. Currently, there is some controversy regarding the influence of *CD14* -260 C/T on the expression of soluble CD14 (sCD14). According to a number of studies, the *CD14* -260 T allele is proposed to increase sCD14 production, which leads to increased serum levels of sCD14 [29], [30], [66], [67]. In contrast, in subjects with *H. pylori* infection elevated sCD14 levels have been associated with the *CD14* -260 CC genotype [52]. Furthermore, others argue that this polymorphism has no effect on transcription [68]. If the *CD14* -260 CC genotype indeed increases sCD14 serum levels, it could explain why the T allele seems to be a protective factor as shown in our case-control study and meta-analysis. Since the current evidence regarding the influence of *CD14* -260 C/T on the expression of soluble CD14 (sCD14) is conflicting, more functional studies are required to clarify this issue.

As *H. pylori* is known to be the main risk factor for GC, we also examined the potential interaction between *H. pylori* and *TLR2*, *TLR4* and *CD14* polymorphisms in the development of GC. Our ethnic Chinese case-control study and meta-analysis showed that selected polymorphisms do not increase the risk of *H. pylori* infection. Furthermore, additional analyses in our ethnic Chinese population showed that the presence of these polymorphisms and *H. pylori* infection increases dramatically the risk of GC in these individuals. Not only *H. pylori*-infected subjects harbouring the *TLR4* rs11536889 C allele showed the highest risk of all but also *H. pylori*-infected individuals harbouring the *TLR2* -196 to -174 deletion allele were shown to be more susceptible to GC, a phenomenon that is not observed if the bacterium is absent. In contrast, the risk of GC in *H. pylori*-infected individuals harbouring the *CD14* -260 T allele, which was shown to be a protective factor in our initial analyses, was not magnified or reduced in the presence of *H. pylori*. Overall, these findings are consistent with our hypothesis that given that *H. pylori* is initially targeted by the TLR signalling pathway, polymorphisms in this branch of the innate immune system could modulate the direction and magnitude of the host response against the infection, explaining why only approximately 1% of *H. pylori*-infected individuals develop GC.

The results obtained in our meta-analysis differ from those presented by the other two available meta-analyses addressing the association between *TLR4* polymorphisms and risk of cancer [27], [31]. From a more generalized perspective, Jing et al. found that *TLR4* Asp299Gly and Thr399Ile increase the risk of gastrointestinal cancers (combining oesophageal, gastric and colorectal cancers) showing ORs of 1.64 (95% CI: 1.02–2.64) and 2.01 (95% CI: 1.40–2.89), respectively [31]. In the context of GC alone, Zhang et al. [27], who attempted to address the association between *TLR4* Asp299Gly and this pathology, showed an OR of 1.85 (95% CI: 1.06–3.22). However, the authors included a study in their meta-analysis that did not comply with the inclusion criteria of our meta-analysis since the subjects recruited in that case-control study did not suffer from GC but a “GC phenotype” characterized by corpus-predominant gastritis or pangastritis [69]. Comparable to the meta-analysis by Jing et al. [31], Zhang et al. showed that *TLR4* Thr399Ile is associated with cancer in general (combining gastric, cervical, colorectal, gallbladder and prostate cancers) showing an OR of 1.81 (95% CI: 1.18–2.77) [27]. In addition, Zhang et al. failed to show an association between *TLR4* rs11536889 and risk of cancer after analysing prostate, gastric and hepatocellular cancers as a group [27]. Thus, the differences between our meta-analysis and the mentioned meta-analyses are due to the types of cancer analysed and the studies included in the analyses.

Our meta-analysis has some limitations. First, publication bias assessment suggested the possibility that studies addressing the association between *CD14* -260 C/T and *TLR4* Asp299Gly polymorphisms and risk of GC that showed negative results, were not published. Nevertheless, asymmetry tests have limited power when the number of included studies in a meta-analysis is small [70]. Although an extensive literature search was performed for this meta-analysis, it is always difficult to measure the extent of unpublished data. Second, the control groups were not homogeneous since both healthy individuals and non-cancer patients (e.g. individuals presenting gastritis, gastric ulcers and peptic ulcers) were included in some of the studies incorporated in the GC meta-analysis. Third, since sensitivity analysis of *TLR2* -196 to -174del, *TLR4* Thr399Ile and *CD14* -260 C/T provided different results compared to the general analysis, these results must be taken with discretion.

In conclusion, based on our ethnic Chinese case-control study and meta-analysis, *TLR2* -196 to -174del, *TLR4* Asp299Gly, *TLR4* rs11536889 and *CD14* -260 C/T polymorphisms appear to play an important role in the development of GC inferring that polymorphisms in the TLR signalling pathway are clearly involved in gastric carcinogenesis. Continued research efforts directed towards the identification of major associations between molecules involved in innate immunity and *H. pylori*-related GC, and subsequent detection of their functional implications, have the potential to provide novel insights into targeted treatment in genetically-susceptible individuals, and thus, improve primary and secondary prevention of *H. pylori*-related GC, a disease that remains the second-leading cause of cancer-related deaths worldwide.

## Supporting Information

Checklist S1 PRISMA checklist for reporting systematic reviews and meta-analysis.
(DOC)
